# How Group-Based Interventions Can Improve Services for People with Severe Obesity

**DOI:** 10.1007/s13679-019-00348-y

**Published:** 2019-05-06

**Authors:** Dawn Swancutt, Mark Tarrant, Jonathan Pinkney

**Affiliations:** 1 Peninsula Schools of Medicine and Dentistry, Plymouth University, ITTC Building, Plymouth Science Park, Plymouth, PL6 8BX UK; 2 College of Medicine and Health , University of Exeter, Heavitree Road, Exeter, EX1 2LU UK; 3 University Medicine, Level 7, University Hospitals Plymouth NHS Trust, Derriford Road, Plymouth, Plymouth PL6 8DH UK

**Keywords:** Severe obesity, Group-based intervention, Tier 3, Behaviour change, Weight management services

## Abstract

**Purpose of Review:**

Rising demand for specialised “Tier 3” weight management services in England is exceeding capacity, leading many services to offer group-based care programmes. This review considers the organisation of current provision, exploring how group programmes may enhance services and how these could be scaled up for wider implementation.

**Recent Findings:**

Existing group-based programmes mainly focus on providing patients with information and education about their condition. Evidence suggests that groups themselves offer therapeutic benefits beyond this, by underpinning patients’ engagement with programme materials and contributing to wider health and well-being. To maximise these benefits, there is a need to attend to the group processes that emerge in treatment groups which, left unchecked, may limit or even adversely impact programme outcomes.

**Summary:**

Group-based interventions may be of benefit to patients in Tier 3 specialist weight management services, although their format is complex and reliant on facilitators’ expertise.

## Introduction

Globally, it is estimated that the prevalence of obesity has more than doubled in over 70 countries since 1980 and a high body mass index (BMI) has accounted for 4 million deaths during the past 25 years [1]. Between 1993 and 2014, while the prevalence of overweight and obesity increased substantially in England, the prevalence of severe obesity rose most steeply from 1.4 to 3.6% (2.5-fold increase) in women and from 0.2 to 1.8% (9 fold increase) in men [2]. There is what some authors describe as an ‘epidemic’ of obesity [3, 4]. Yet it is not obesity alone that causes such concern. Whereas links between obesity and conditions such as type 2 diabetes are well-established, newer risk estimates for other serious health conditions highlight the impact of this global issue. The recent US Centre for Disease Control Report has highlighted the link between obesity and cancer, estimating 55% of all cancers diagnosed in women being associated with overweight and obesity [5].

This rising prevalence of severe obesity and associated health risks, in conjunction with limited health service resources, means that more innovative solutions are required for the treatment and support of individuals who have this condition. One potential solution may be to use group interventions to support delivery of specialist weight management services. Group-based approaches have been adopted by some of these services across England, but this remains the exception rather than the rule. In this article, we examine the guidelines, current use and potential of moving to a more group-based approach in the delivery of services for people with severe obesity in the National Health Service (NHS) in England.

## Weight Management Service Guidelines in England

In England, four distinct “Tiers” of weight management services are recognised [6]. Tier 1 services take a public health perspective and represent universal behavioural interventions to promote healthy choices throughout the whole population. Tier 2 services are local community weight management services (NHS and commercially run), aimed at supporting weight loss (and weight loss maintenance) in people who are overweight. Most Tier 2 services are run as group-based sessions. Services designed to support patients in the higher BMI categories, either > 40 or > 35 kg/m^2^ with comorbidities, are known as Tier 3 services. These services offer specialist assessment and behavioural and medical treatments and offer the only referral route for patients who go onto bariatric surgical (Tier 4) services. Not all regions have a Tier 3 service available [7].

The National Institute of Clinical Excellence offers guidelines on identification and treatment of people with severe obesity [8]. Multidisciplinary team support is a key requirement for Tier 3 services [8, 9] although it is widely recognised that the structures of these services, and the design of their interventions, vary considerably [6, 10]. While a proportion of people with severe obesity using Tier 3 services choose bariatric surgery, the majority do not. Of the number of people with severe obesity as a whole, it is estimated that in the UK less than 1% of those who could benefit access surgery [11]. Therefore, Tier 3 services need to design and deliver treatment programmes that best meet the needs of both sets of patients, those who go on to surgery and those who prioritise weight loss without surgical intervention.

A range of guidelines are available on the content of weight management services [8, 9, 12, 13]. While this guidance broadly describes the main therapeutic approaches to weight management, there is little detail on the precise design of weight management programmes, in terms of intervention configuration. As we outline later, group-based care potentially offers a valuable means of structuring patients’ engagement with weight management programmes and may fundamentally address psychological and well-being needs related to their condition. As happens for other health conditions, we similarly suggest that “the group” may constitute an important part of the solution [14, 15•].

## Potential Benefits of Group-Based Interventions

From a purely economic perspective, the group format may allow for an efficient use of staff time. For example, 12 patients previously seen individually for 10 1-h sessions (120 staff hours, 10 patient hours) might form one group of 12 attending 20 2-h group sessions (40 staff hours, 40 patient hours), providing benefits of group support and saving up to 80 staff hours with four times more patient contact. Group-based delivery may also reduce waiting times for treatment: delays in access have been widely acknowledged as a key factor affecting service provision, with oversubscription and long waiting lists reported for Tier 3 services, causing delays of many months for patients to access programmes [10].

The group format may also bring therapeutic benefits to patients. In lower weight populations, and in other health conditions, group-based care can be an effective means of delivering treatment interventions [16–18]. A systematic review of evidence on the effectiveness of group interventions for those with chronic conditions, of which diabetes formed the majority, found consistent and promising evidence for a positive effect of group-based care on biomedical outcome measures [16]. Group interventions were found to be more effective in improving HbA1c and systolic blood pressure than traditional care.

In terms of weight loss specifically, group interventions for patients with obesity have been shown to have a greater impact on weight change at 12 months than therapy delivered individually [19]. More recently, a systematic review and meta-analysis of 47 randomised controlled trials (reporting 60 evaluations) for patients with BMI ≥ 25 showed clinically meaningful weight loss at 12 months in interventions focused on diet and physical activity [20•]. However, considerable heterogeneity was observed across studies in this review, both in terms of effect sizes (the mean difference in weight loss varied from − 9.6 to 0 kg) and intervention design, delivery and control comparison. Thus, despite some encouraging evidence that group interventions can effectively support weight management programmes, actually very little is known about how groups should be organised to optimise their effectiveness.

## How Weight Management Services Currently Use Group Sessions

### National Mapping of Service Provision in England

A 2015 national mapping survey of NHS weight management services explored service provision in England. At the time of the survey, both local authorities and Clinical Commissioning Groups funded specialist services. Forty-three service representatives responded to the survey, with a geographical coverage of 13% (19/152) of local authorities and 12% (26/209) of Clinical Commissioning Groups. The survey identified referral routes and entry criteria, service details, costs, exit routes and barriers to commissioning services. Some basic information on use of groups was collected with the majority of services described using group programmes as well as one-to-one support to deliver their multicomponent interventions [10]. However, the low response rate makes it unclear how representative these patterns are of wider UK service provision.

Despite some guidance advocating the use of group programmes in the management of patients with obesity [12, 19], and a recognition that group-based support is a predictor of longer-term weight loss [21•], practical information supporting the use of groups is lacking—a situation likely reflecting the limitations of the literature noted above. As demand for weight management services for severe obesity increases, it is clear that (i) more services may turn to using groups as part of the care pathway but (ii) the limited evidence currently available means that services have few resources to help them offer patients an effective, evidence-based treatment for their condition.

### Scoping Review of How Tier 3 Services Currently Use Groups

Between March and June 2016, we contacted members of the Association for the Study of Obesity UK (UK ASO) to explore how groups were currently being used in their Tier 3 services [22]. Following an initial e-mail to 22 ASO members, inviting feedback from those using a group format, we used snowball sampling to expand our links to other Tier 3 providers. We followed this up with telephone conversations to gather further details. These conversations were loosely structured on Borek et al.’s (2015) guide to reporting group-based healthcare interventions and asked about group organisation (number of sessions, duration, etc.), content, participants, facilitators and style of programme [23]. We obtained a detailed breakdown from nine Tier 3 services, eight in England and one in Wales. Throughput for services varied from approximately 100 to 700 patients per year. We were purposively looking for variation in group use, which even in this small sample we found varied widely. Table 1 summarises the configurations of these group-based programmes.

**Table 1:** Group-based programme formats currently in a sample of Tier 3 weight management clinics in the UK [21]

Programme identifier	No. of group sessions offered	Session duration (min)	Regularity	Length of programme	Group size (*n*)	Participant may bring a companion?	Session delivered by:
1	1	60	One-off	6	6–8	Yes	Nurses and dietician
2	12	60	Weekly	6	6–8	No	Physical activity lead
3	5	60	One-off	12	3–20	Not generally	Varies according to purpose
4	8	60–90	Weekly/monthly	6	5–12	Carers if required	Dietician or counsellor
5	8	120	Fortnightly	6	Max 18	No	Dietician and physiotherapist
6	24	40	Weekly	6	12–14	No	Dietician, nutritionist or physical activity lead
7	28	90 and 60	Weekly/fortnightly/monthly	24	Max 15	No	Nurse specialist, dietician or psychologist
8	4	90	Monthly	6	Max 8	No	Dietician
9	7	90	Monthly	6	Max 8	No	Dietician and psychologist

Not only were the objectives (motives underpinning weight loss achievement, such as behaviour change theory, educational and empathetic focus) and forms of delivery of group-based interventions highly variable but also too were factors like intervention content, session locations, intensity, duration, patient selection, starting group sizes and the professional backgrounds and training of group facilitators [22]. We observed a wide range in the number of group sessions offered, from one to 28 (with additional group support led by peer leaders). Session duration was often 1 h, but ranged from 40 min to 2 h; longer sessions included an exercise component. Frequency of group meetings ranged from weekly to monthly, and some varied in frequency—initially weekly then decreasing to monthly. The shortest programme reported was 6 months, referring to when the final weight measurement is recorded, and the longest being 24 months. Group sizes were also variable, with some including partners or carers.

We know from this scoping work that many different forms of group-based and one-to-one care, or combinations of the two, are in use by Tier 3 services. Furthermore, the term ‘group-based’ care was used loosely by responding services to describe quite a wide range of activities. These included short sessions on entry to a programme, mainly used for detailed didactic information giving about lifestyle change, what Drum et al. [24] have termed ‘psychoeducational groups’. Notably absent from this review were any approaches to group intervention that emphasised the potential therapeutic benefits to patients of being in a group itself. It is to this potential that we now turn.

### Why the ‘Group’ Element of Group Treatment Is Important

As we outline below, research suggests that group-based activities have a wide range of potential benefits beyond being a pragmatic delivery mode for existing programme content (e.g., psychoeducational groups). Central to the conclusions from this research is the assertion that groups can serve as a powerful basis supporting intervention delivery (and therefore behavioural change) and also contribute to members’ wider well-being. However, this potential can only be realised to the extent that patients come to experience a meaningful sense of social connection to other patients within the treatment group, that is, to the extent that they experience *shared social identity*. In order to harness this therapeutic potential, it therefore becomes important for those responsible for delivering group interventions to nurture a treatment environment that encourages patients to see themselves *as group members*.

The potential health benefits of developing a shared social identity as a member of a treatment group have been demonstrated across different health conditions, including obesity [25, 26]. Our qualitative investigation of a group-based Tier 3 programme showed how treatment group social identity was regarded by patients as a key mechanism structuring their engagement with intervention materials and progression through the group programme [27•]. Specifically, patients reported that a shared social identity (i) underpinned their ability to engage with the programme’s dietetic content (i.e., group-based learning) and (ii) facilitated access to the psychological resources needed to put this learning into practice through initiating changes to behaviour. On one hand, the social support derived from other group members helped patients realise that they were “not alone” in dealing with their health issues; and on the other hand, by learning about others’ experiences (e.g., successes in weight loss), patients experienced increased self-efficacy that they felt allowed them to pursue their individual change goals.

Patient dropout from weight management programmes has been shown to be greatest at the start of a group programme [28], suggesting a need to attend to participants’ early experiences within the group in order to help shape a positive shared social identity that allows for the group to become a therapeutic resource. Indeed, a recent study has highlighted some of the consequences of *not* attending to the processes that shape social identity formation. In their investigation of a group-based weight loss intervention for people with obesity in the USA, Nackers et al. (2015) found that perceived conflict within the treatment group was associated with poorer patient adherence to the intervention (completion of dietary intake and physical activity logs) and also lower levels of attendance at the group sessions [29•]. Moreover, group conflict predicted lower weight loss at 6 months.

### A New Agenda for Research into Group-Based Behavioural Interventions for Tier 3 Services

Despite evidence highlighting the importance of group processes in structuring health outcomes, actually little is known about how to construct group interventions for people with severe obesity that deliver on the therapeutic potential offered by establishing shared social identity amongst patients. Our research is starting to understand the processes by which groups can be assembled in clinical settings in order to capitalise on their clear potential [27•, 31, 32]. From this research, we can outline five key principles we suggest need to be considered when designing group-based behavioural interventions for people with severe obesity. This list is not exhaustive and there is scope to extend the evidence base that underpins them.

1. Making ‘the group’ psychologically meaningful for patients

There is a need to understand how to effectively manage group processes that impact delivery of Tier 3 interventions, including how to build and maintain shared social identity amongst patients. Evidence-based guidelines are needed for delivering wider intervention content focused on individual behavioural change in the group format. It is unspecified how existing techniques for supporting weight management (e.g., CALO-RE; Michie et al. 2011 [30]) should be adapted for use in group interventions for this patient population. Some of our work with other health conditions is starting to explore how to do this in practice [31, 32].

2. The patients

Second, people with severe obesity experience significant psychological problems (including low self-esteem, anxiety and depression and stigmatisation [3]). It is important to understand (i) how such factors might impact on individual patients’ ability, or readiness, to engage with group-based interventions and particularly how these might inhibit the formation of shared social identity. Relatedly, clearly some patients may not wish to be part of a group. How can such patients best be supported in services that are organised around group-based care? We suggest that early engagement (pre-intervention) with such patients may be needed in order to alleviate any anxieties they may have about joining a new group. Other options may include “buddying-up” with past patients or other incoming patients in order to start to build familiarity with each other prior to joining a group.

3. The group facilitators

Third, group leaders play a critical role in shaping social identities [33]. Group leaders (treatment group facilitators) “set the scene” for Tier 3 groups and as such can help create an environment that helps realise the therapeutic potential of the group. The skills needed to manage a treatment group are likely to be different from those needed in one-to-one intervention [34]. What are these skills and how can they be taught to group facilitators? The development of practice guidelines could help establish a treatment culture that prioritises the importance of addressing group processes in intervention settings.

4. Complexity

Fourth, group interventions are rarely straightforward or simple and, actually, are usually quite complex in nature. They can be composed of several interacting components: complexity may arise due to the number of outcomes the intervention is focused on changing (e.g., dietary behaviour and physical activity), variability in the target population (comorbidities, pre-existing psychological conditions) or the number of elements of the intervention itself [35]. Moreover, as Hoddinott (2010) notes, theories that are thought to be helpful in behaviour change for individual use are often assumed to be generalisable to group settings, but this is not necessarily the case: health improvement in groups depends on complex adaptive social processes, where a wider set of interactions takes place [36]. The analysis we have presented here clearly supports this argument. A challenge for designers of group-based interventions, therefore, is to account for and disentangle such complexity.

5. Reporting transparency

A final issue concerns the reporting of research (or indeed service) evaluations of current Tier 3 provision. We acknowledge the valuable contribution that reporting details of Tier 3 interventions provide, such as those in Norfolk, Liverpool, Glasgow and Birmingham amongst others [37–40]. However, in general, the reporting of group interventions has been highlighted as problematic [23]. Thus, even when the effectiveness of group-based weight management interventions has been demonstrated [20•], poor reporting (either of intervention components, theoretical bases or both) limits their translational value and makes comparisons between different programmes difficult, if not impossible [21•]. Tools now exist to support the transparent reporting of group interventions [23] and we recommend their use in research.

## Conclusion

The growth of Tier 3 specialist weight management services in England has resulted in the adoption of a diverse range of formats, including an increasing reliance on group-based intervention. While evidence is currently lacking on the optimal format of such interventions, studies in other settings suggest that group intervention can be at least as effective as one-to-one care and may offer significant wider therapeutic benefits. However, group-based programmes are inherently complex and not without their challenges, including the need for trained facilitators to harness and manage group processes in ways that encourage patients to form meaningful psychological connections with each other and shared social identity. By bringing attention to these issues, we hope that the field of obesity research can contribute a better understanding of the mechanisms for effective group-based behavioural intervention and in doing so can improve patients’ experiences and outcomes of Tier 3 services.
